# Effect of Reaction Time of TGase on the Water-Holding Capacity and Gel Properties of Reduced-Fat and Reduced-Sodium Chicken Meat Batters

**DOI:** 10.3390/gels11110848

**Published:** 2025-10-23

**Authors:** Dongyang Zhu, Ke Xu, Zhuangli Kang, Bo Luo, Kun Fang

**Affiliations:** 1 Henan Key Laboratory of Tea Plant Biology, College of Tea and Food Science, Xinyang Normal University, Xinyang 464000, China; aa3154787371@163.com (K.X.); luobo2011@163.com (B.L.); fangkun@xynu.edu.cn (K.F.); 2 Dabie Mountain Laboratory, Xinyang 464000, China; 3 College of Tourism and Cooking, Yangzhou University, Yangzhou 225127, China; kzlnj1988@163.com

**Keywords:** TGase, reaction times, water-holding capacity, gel properties, chicken meat batter

## Abstract

In this paper, the effects of TGase reaction times (0, 6, 12, 18, and 24 h) at 4 °C on the solubility, emulsion stability, cooking yield, gel properties and water distribution of reduced-fat and reduced-sodium chicken meat batter were studied. The results showed that the reaction time had a significant effect on the water fluidity and quality characteristics of reduced-fat and reduced-sodium chicken meat batter. The solubility, cooking yield and water-holding capacity of salt-soluble proteins initially increased then decreased with extended reaction time, reaching maximum values of 65.50%, 96.13% and 96.00%, respectively, at 12 h. The emulsifying stability and textural properties initially increased, then decreased with extended reaction time (*p* < 0.05), achieving optimal levels at 12 h. In contrast, the initial relaxation time of T21 and T22 initially decreased (*p* < 0.05) and then increased (*p* < 0.05) with longer reaction times; the minimum values were 12 h, especially the free water decreased from 17.97% to 6.69%, consistent with the finding on water-holding capacity and gel properties. In conclusion, the reaction time of the TGase affected its effect on improving the gel effect of reduced-fat and reduced-sodium chicken meat batter, and the best effect was achieved at 12 h.

## 1. Introduction

From a health and nutrition perspective, meat and meat products have long been considered primary sources of protein, vitamins, and minerals, supplying essential amino acids required by the human body [1]. During processing, salt, fat, soy protein, and other additives are commonly used to enhance product quality and flavor. Salt improves taste and promotes flavor compound formation, thereby extending shelf life and enhancing flavor [2,3]. Fat is essential for reducing cooking losses and improving water and oil retention, as well as enhancing juiciness [4]. Traditional emulsified meat products typically contain high levels of fat (20–30%) and salt (2–3%); however, high intake of salt and fat has been linked to increased risks of cardiovascular diseases, osteoporosis, kidney disease, and diabetes [5,6,7]. With growing public awareness of healthy eating, reducing salt and fat in meat products while maintaining quality has become a key research focus in the meat industry [8]. Sodium chloride promotes the formation of a dense gel network by influencing protein swelling in gel-type meat products [9,10]. Reducing salt levels can impair the solubility of salt-soluble proteins, leading to decreased water and oil retention after heating, which negatively affects product quality [11]. Similarly, fat content influences the stability and hardness of gel-type meat products. Lowering fat may result in reduced slicing performance, texture, and color [12]. Therefore, developing strategies to reduce salt and fat while maintaining or improving meat product quality remains a major challenge [13].

In recent years, modifying protein cross-linking through natural or induced methods has altered the texture of meat products, including hardness, cohesion, and springiness [14]. Transglutaminase (TG) catalyzes acyl transfer reactions, forming ε-(γ-Glu)-Lys peptide bonds within and between proteins using glutamine residues as acyl donors. The ability of TGase to improve the functional properties of meat proteins is well documented. For example, TGase enhances the physicochemical properties of frankfurters and meat batters [15,16]. Polysaccharides are frequently employed as functional materials to replace fats in low-fat emulsified meat products, thereby enhancing product quality. Sodium alginate is widely used in emulsions and restructured meat products due to its thickening, stabilizing, and gelling properties. It can improve juiciness, texture, color, and flavor [17,18]. Our previous study found that replacing 50% pork back fat with sodium alginate solution can enhance water-holding capacity and texture while reducing fat and energy content in frankfurters [19]. Despite extensive research on TGase in meat products, few studies have explored the impact of TGase reaction time on the quality of reduced-fat and reduced-sodium chicken meat batter containing sodium alginate. This study examines the effects of varying TGase reaction times (0 h, 6 h, 12 h, 18 h, 24 h) on salt-soluble protein solubility, cooking yield, emulsion stability, water-holding capacity, color, texture, and moisture distribution and mobility. The findings aim to clarify how TGase reaction time affects product quality and provide a theoretical basis for developing healthier, reduced-fat and reduced-sodium gel-type meat products.

## 2. Results and Discussion

### 2.1. Solubility

**Figure 1:**
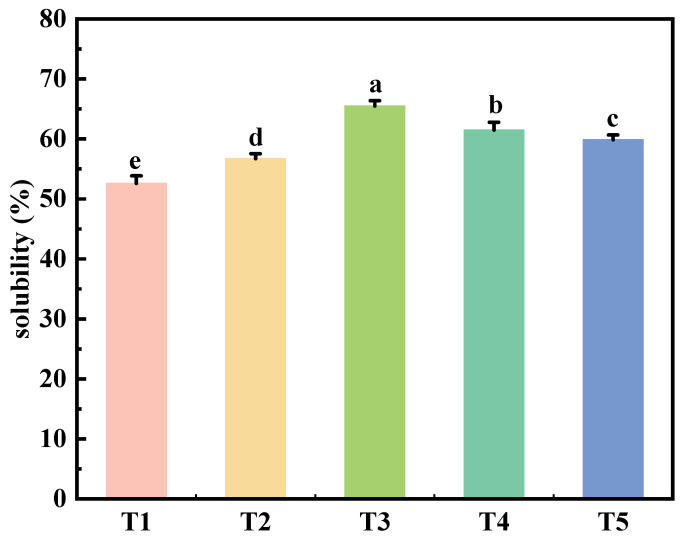
Effect of reaction time on the solubility of reduced-fat and reduced-sodium chicken meat batter with TGase. T1: reaction at 4 °C for 0 h; T2: reaction at 4 °C for 6 h; T3: reaction at 4 °C for 12 h; T4: reaction at 4 °C for 18 h; T5: reaction at 4 °C for 24 h. Each value is the mean ± SE, n = 4. a–e Different parameter superscripts indicate significant differences (*p* < 0.05).

Figure 1 illustrates the impact of TGase reaction time on the solubility of salt-soluble proteins in reduced-fat and reduced-sodium chicken meat batter. The results indicated that extending the TGase reaction time significantly enhances (*p* < 0.05) the solubility of salt-soluble proteins. As the TGase reaction time increased from 0 to 24 h, the solubility of salt-soluble proteins initially rose and then declined. Specifically, when the reaction time was extended from 0 to 12 h, there was a significant increase in solubility (*p* < 0.05), with the maximum solubility observed at 12 h. This phenomenon can be attributed to TGase and salt concentrations may accelerate the degradation of undissolved myofibrillar proteins and enhance their solubility as the TGase reaction time increases. The formation of intramolecular and intermolecular ε-(γ-glutamyl) lysine (G-L) cross-links through the acylation of protein lysine residues leads to the development of a stable protein network, thereby improving the solubility of salt-soluble proteins. Previous similar studies have found that TGase could enhance the cross-linking between protein molecules, alter the protein network structure, and improve the solubility of muscle protein [20,21]. However, when the TGase reaction time exceeded 12 h, the solubility of salt-soluble proteins decreased. This decline might result from the number of cross-linked proteins increasing, the depletion of free lysine and lysine residues in the reaction system, leading to water acting as an acyl receptor and glutamine residues utransglutaminasendergoing deamidation to form glutamic acid, which reduced protein solubility [15].

### 2.2. Emulsification Stability

As illustrated in Table 1, significant differences (*p* < 0.05) were observed in the total fluid, water, and fat released of reduced-fat and reduced-sodium chicken meat batter gel under varying TGase reaction times. Initially, as the reaction time increased from 0 h to 12 h, these exudation rates gradually decreased, reaching their minimum at 12 h. This suggested that the emulsification stability of the chicken meat batter improved with extended reaction time within this period. The prolonged reaction time allowed TGases sufficient interaction with salt-soluble protein substrates, enhancing inter- and intra-protein interactions [22]. Consequently, a denser gel network structure was formed during thermal processing, effectively binding water molecules and oils within the gel matrix, thereby improving emulsion stability [23]. However, when the reaction time was extended beyond 12 h to 24 h, the total fluid, water and fat released all showed an increasing trend, indicating a gradual weakening of emulsification stability. This might be attributed to the reduced solubility of salt-soluble proteins (Figure 1), which decreased with excessive TGase action time, leading to diminished emulsification stability.

### 2.3. Cooking Yield

**Figure 2:**
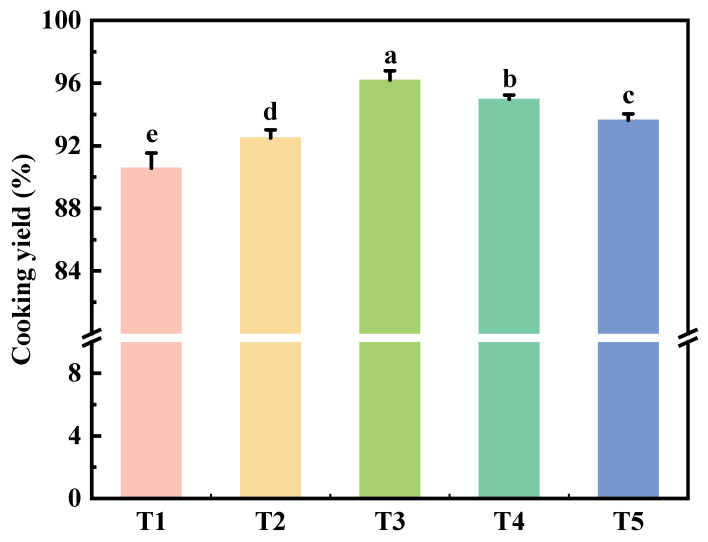
Effect of reaction time on cooking yield of reduced-fat and reduced-sodium chicken meat batter with TGase. T1: reaction at 4 °C for 0 h; T2: reaction at 4 °C for 6 h; T3: reaction at 4 °C for 12 h; T4: reaction at 4 °C for 18 h; T5: reaction at 4 °C for 24 h. Each value is the mean ± SE, n = 4. a–e Different parameter superscripts indicate significant differences (*p* < 0.05).

As shown in Figure 2, different TGase reaction times significantly affected (*p* < 0.05) the cooking yield of reduced-fat and reduced-sodium chicken meat batter. The cooking yield increased significantly (*p* < 0.05) from 0 h to 12 h, peaking at 12 h, but subsequently decreased (*p* < 0.05) as the reaction time extended from 12 h to 24 h. This trend aligns with changes in salt-soluble protein solubility (Figure 1) and emulsification stability (Table 1). Shorter reaction times result in insufficient cross-linking between proteins, leading to a less stable gel structure and lower cooking yield [24]. Conversely, as the reaction time increased up to 12 h, enhanced cross-linking led to a more compact gel structure, improving the cooking rate. Beyond 12 h, however, excessive cross-linking hindered the formation of a stable gel network, reducing cooking efficiency. Similar study [25] found that myosin in pork serves as an effective substrate for TGases, promoting the formation of G-L (Glu-Lys) bonds, which strengthens the protein structure and improves water-holding capacity and cooking yield [26].

### 2.4. Water-Holding Capacity

**Figure 3:**
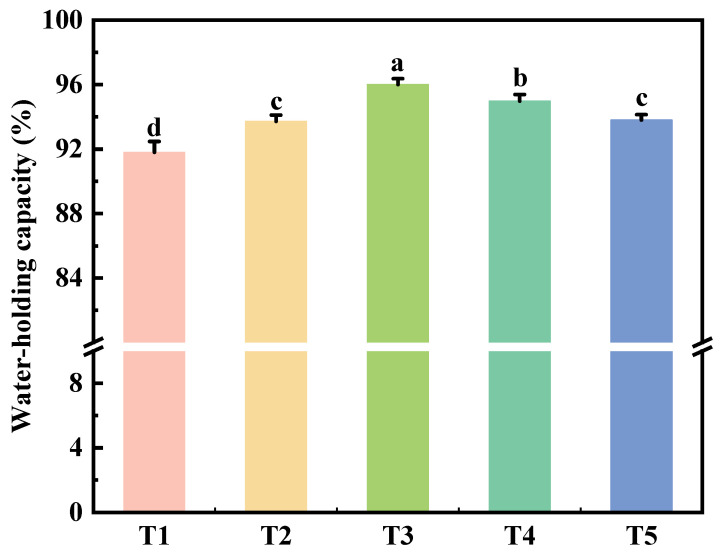
Effect of reaction time on water-holding capacity of reduced-fat and reduced-sodium chicken meat batter gel with TGase. T1: reaction at 4 °C for 0 h; T2: reaction at 4 °C for 6 h; T3: reaction at 4 °C for 12 h; T4: reaction at 4 °C for 18 h; T5: reaction at 4 °C for 24 h. Each value is the mean ± SE, n = 4. a–d Different parameter superscripts indicate significant differences (*p* < 0.05).

Water-holding capacity (WHC) refers to the ability of proteins to absorb and retain water through protein-water interactions [27]. Figure 3 illustrates the impact of varying TGase reaction times on the WHC of reduced-fat and reduced-sodium chicken meat batter gel. Initially, as the TGase reaction time increased from 0 h to 12 h, the WHC of the chicken meat batter significantly improved (*p* < 0.05), reaching its peak at 12 h. This enhancement is attributed to the formation of a robust gel network, which requires adequate cross-linking between protein molecules [28]. Extended reaction times facilitate protein aggregation and cross-linking, thereby improving the springiness of muscle protein gel and forming a dense protein–protein gel network capable of retaining substantial amounts of water [15]. However, when the reaction time was extended beyond 12 h to 24 h, the WHC of the chicken meat batter decreased. This decline may be due to excessive cross-linking between protein molecules, which can disrupt the gel network structure, leading to instability and reduced WHC. The primary reason for this phenomenon is that deamidation of glutamine residues in TGases increases polymerization of protein chains, producing a strong gel structure [29]. Prolonged TGase reaction times can cause excessive cross-linking, further destabilizing the gel network and reducing water retention.

### 2.5. Color

As shown in Table 2, with increasing TGase reaction time, significant differences were observed in the color parameters of reduced-fat and reduced-sodium chicken meat batter gel (*p* < 0.05). Compared to T1, the L* value initially increased and then decreased, while the a* value significantly decreased (*p* < 0.05), and the b* value remained unchanged (*p* > 0.05). Specifically, as the TGase reaction time increased from 0 h to 12 h, the L* value of the chicken meat batter showed an upward trend, peaking at 12 h. Conversely, when the reaction time extended from 12 h to 24 h, the L* value declined. This trend may be attributed to the coprice bond induced by TGase, which enhances the gel network structure and improves WHC, thereby increasing light reflection and the L* value within the first 12 h [30]. As the reaction time continued to increase, over-cross-linking of proteins disrupted the gel network, reducing WHC and consequently lowering the L* value. The b* value did not change significantly with increasing TGase reaction time (*p* > 0.05), indicating that TGase reaction time had no significant effect on yellow-blue reflection and absorption [31].

### 2.6. Texture Characteristics

Table 3 shows how TGase reaction time affects the hardness, springiness, cohesion, and resilience of reduced-fat and reduced-sodium chicken meat batter gel. Compared to T1, these texture properties initially increased (*p* < 0.05) and then decreased (*p* < 0.05) with longer reaction times, peaking at 12 h. This is because TGase promotes the formation of ε-(γ-glutamylamino) lysine covalent bonds, which help create a strong three-dimensional gel network through molecular cross-linking [16], so previous studies reported that TGase-treated muscle protein gels became harder [15,32]. However, extending the reaction time beyond 12 h caused hardness, springiness, and chewiness to decline (*p* < 0.05). This may be due to side reactions when TGase acts for too long, such as the hydrolysis of glutamyl residues into glutamic acid, which changes the isoelectric point of myofibrillar proteins and weakens gel texture [33].

### 2.7. LF-NMR

LF-NMR is a non-destructive and rapid method for analyzing moisture distribution and mobility in meat gel products [34,35]. Table 4 presents the initial relaxation time and peak ratio of cooked reduced-fat and reduced-sodium chicken meat batter gel under different TGase reaction times. Three relaxation populations were identified at approximately 0–10 ms, 10–100 ms, and 100–1000 ms, corresponding to T_2b_ (bound water), T_21_ (non-mobility water), and T_22_ (free water), respectively [36,37,38]. The T_2b_ relaxation time remained unchanged (*p* > 0.05) with prolonged reaction time. In contrast, the T_21_ and T_22_ relaxation times first decreased (*p* < 0.05) and then increased (*p* < 0.05), reaching minimum values at 12 h. Previous studies suggest that shorter T_2_ relaxation times indicate tighter water binding and lower mobility [15,39]. This implies that bound water mobility was unaffected by reaction time, while non-mobility and free water mobility first decreased and then increased, with the lowest values at 12 h, consistent results of water-holding capacity. Similarly, no significant change (*p* > 0.05) was observed in the P_2b_ peak ratio. The P_21_ peak ratio first increased (*p* < 0.05) and then decreased (*p* < 0.05), peaking at 12 h, while P_22_ showed the opposite trend, reaching a minimum at 12 h. These results indicate that the batter achieved the highest non-mobility water content and lowest free water content by creating covalent cross-links within the protein gel, which makes the network more rigid and resistant to breakdown after 12 h of reaction at 4 °C, consistent with the findings on gel properties.

## 3. Conclusions

The reaction time of TGase had a significant effect on the quality characteristics of reduced-fat and reduced-sodium chicken meat batter gel. With the increase in reaction time, the salt-soluble protein solubility, emulsion stability, cooking yield, water retention, texture properties, and the proportion of non-mobility water of reduced-fat and reduced-sodium chicken meat batter gel all increased first and then decreased, and all reached the maximum value at 12 h. The gel quality decreased with increasing reaction time (12–24 h). Conversely, the non-mobility water and free water relaxation times first decreased and then increased, reaching minimum values at 12 h. Therefore, the TGase reaction time has a significant difference in improving the quality of reduced-fat and reduced-sodium chicken meat batter. Choosing an appropriate reaction time can significantly improve the gel quality of reduced-fat and reduced-sodium chicken meat batter, while too long a reaction time may damage the formation of the gel network and cause the quality to decline. In summary, the TGase reaction time of 6–24 h can significantly improve the quality of reduced-fat and reduced-sodium chicken meat batter, and the effect of 12 h is the best. This approach seamlessly integrates with existing meat processing lines, substantially reducing microbial spoilage risks during storage in the temperature danger zone. It imparts exceptional textural characteristics, holding promise for advancing the development of premium products.

## 4. Materials and Methods

### 4.1. Materials and Reagents

Fresh chicken breast meat and fresh pork back fat, along with spices and food-grade sodium chloride, were purchased from local farmers’ markets in Xinyang, China. Visible fat and connective tissue were removed from the meat. Both meat and back fat were ground using a 6 mm plate. The meat was vacuum-packed in polyethylene bags and stored at −40 °C for up to two weeks. The back fat was further ground using a 2 mm plate two h before use. Food-grade sodium alginate was provided by Shanghai Xintai Industrial Co., Ltd. (Shanghai, China), and TGase (1% TGase, 99% maltodextrin) was supplied by Jiangsu Taixing Dongsheng Food Biotechnology Co., Ltd. (Taixing, China).

### 4.2. Preparation of Reduced-Fat and Reduced-Sodium Chicken Meat Batter

To prepare the sodium alginate solution, 10 g of sodium alginate was dissolved in 300 g of distilled water at room temperature. The solution was stirred for 60 min at 1000 rpm using an EMS-19 magnetic stirrer (Tianjin ONuo Instrument Co., Ltd., Tianjin, China), then cooled to 4 °C. Based on the formula in Table 5, 200 g of ground meat was placed into a chopping machine (Stephan nmc5, Hameln, Germany). Sodium chloride, TGase, spices, and one-third of the ice water were added. The mixture was blended for 30 s at 1500 rpm, rested for 3 min, and then the ground back fat, sodium alginate solution, and remaining ice water were added. This mixture was blended again for 30 s at 1500 rpm and rested for another 3 min. Finally, it was chopped at 3000 rpm for 60 s, ensuring the temperature remained below 8 °C.

The chicken meat batter (35 g) was transferred into a 50 mL centrifuge tube and centrifuged at 1500× *g* for 10 min at 4 °C to remove air bubbles. It was then stored at 4 °C for different durations: 0 h (T1), 6 h (T2), 12 h (T3), 18 h (T4), and 24 h (T5). After storage, heat the centrifuge tubes containing chicken meat batters directly in an 80 °C water bath for 30 min (central temperature 72 °C) and cool overnight at 4 °C.

### 4.3. Determination of Solubility of Salt-Soluble Protein

Ten grams of raw chicken meat batter were mixed with 50 mL of pre-cooled (2–4 °C) phosphate-buffered solution (20 mM, pH 7.0). The mixture was homogenized for 90 s using a T25 high-speed homogenizer (IKA Equipment, Staufen im Breisgau, Germany) at 15,000 rpm. The homogenate was then centrifuged at 10,000× *g* for 30 min at 4 °C. The supernatant was collected. The solubility of salt-soluble proteins was determined using the BCA method, whilst the total protein concentration in raw minced chicken was measured by the Kjeldahl method. Protein solubility was calculated as the ratio of salt-soluble protein concentration to total protein concentration.

### 4.4. Determination of Emulsification Stability

The method of emulsification Stability was according to Liu et al. [29]. Thirty-five grams of chicken meat batter were placed in a centrifuge tube and centrifuged at 1500× *g* for 10 min at 4 °C to remove air bubbles. The sealed tube was heated in a water bath at 80 °C for 30 min. After heating, the tube was dried, opened, and inverted on a plate for approximately 50 min. Total exudate rate was calculated as the percentage of exudate relative to the initial sample weight. After drying the exudate at 105 °C for 16 h, fat exudate rate was determined as the percentage of residual dry matter relative to the sample weight. Water exudate rate was calculated as the percentage of released water relative to the sample weight.

### 4.5. Determination of Cooking Yield

The raw chicken meat batter (approximately 35 g) was transferred into a 50 mL centrifuge tube, removing air bubbles. The batter was heated in a water bath at 80 °C for 30 min (central temperature 72 °C) at different reaction times and cooled overnight at 4 °C. The chicken meat batter was equilibrated at room temperature for 2 h. Surface moisture was removed with absorbent paper. Cooking yield (%) was calculated as follows:

Cooking yield (%) = (Weight of chicken meat batter after cooking/Weight of chicken meat batter before cooking) × 100%

### 4.6. Determination of Water-Holding Capacity

The method of water-holding capacity was according to Wang et al. [17]. Approximately 10 g of chicken meat batter gel (W0) was centrifuged for 15 min at 10,000× *g*. Surface moisture was removed with absorbent paper, and the samples were weighed (W1). Water-holding capacity (%) was calculated as follows:

Water-holding capacity (%) = (1 − (W0 − W1)/W0) × 100%

### 4.7. Color Measurement

After overnight refrigeration at 4 °C, the chicken meat batter was warmed to room temperature for 2 h. Surface moisture was removed with absorbent paper. Cylindrical samples (1 cm in height and diameter) were cut and analyzed using a CR-400 colorimeter (Konica Minolta, Inc., Tokyo, Japan). Standard white board values were L* = 97.32, a* = −0.21, b* = 1.71.

### 4.8. Determination of Texture Characteristics

The method of texture characteristics was according to Kang et al. [18]. Chicken meat batter was refrigerated overnight at 4 °C and then warmed to room temperature for 2 h. Surface moisture was removed with absorbent paper. Samples were cut into cylinders (2.5 cm height) and analyzed usingupp a TA.XT Express texture analyzer. Parameters included 50% compression, 5 g trigger force, pre-test speed of 2.00 mm/s, test speed of 2.00 mm/s, and post-test speed of 3.00 mm/s. Hardness (N), springiness, cohesiveness, and chewiness (N·mm) were recorded.

### 4.9. Low Field Nuclear Magnetic Resonance (LF-NMR)

Approximately 2 g of the cooked batter was placed in a 15 mm glass tube and inserted in the NMR probe of a Pulsed NMR analyzer (NMI20-040 V-1, Niumag Electric Corporation, Shanghai, China) operated at a resonance frequency of 22 MHz. The parameters used were as follows: 32 repeated scans with a time interval of 110 ms.

### 4.10. Statistical Analysis

Each experiment was repeated four times using different materials. Data were expressed as mean ± SE. One-way ANOVA was performed using SPSS v.26.0. Differences were considered significant at *p* < 0.05 using Duncan’s Multiple Range Test.

## Figures and Tables

**Table 1:** Effect of reaction time on emulsifying stability of reduced-fat and reduced-sodium chicken meat batter gel with TGase.

Sample	Total Fluid Released (%)	Water Released (%)	Fat Released (%)
T1	17.54 ± 0.25 ^a^	16.29 ± 0.19 ^a^	1.25 ± 0.06 ^a^
T2	15.60 ± 0.28 ^b^	14.51 ± 0.27 ^b^	1.09 ± 0.05 ^b^
T3	12.18 ± 0.13 ^e^	11.35 ± 0.08 ^e^	0.84 ± 0.05 ^d^
T4	13.47 ± 0.36 ^d^	12.57 ± 0.35 ^d^	0.90 ± 0.05 ^d^
T5	14.38 ± 0.16 ^c^	13.39 ± 0.15 ^c^	0.99 ± 0.04 ^c^

T1: reaction at 4 °C for 0 h; T2: reaction at 4 °C for 6 h; T3: reaction at 4 °C for 12 h; T4: reaction at 4 °C for 18 h; T5: reaction at 4 °C for 24 h. Each value is the mean ± SE, n = 4. a–e Different parameter superscripts indicate significant differences (*p* < 0.05).

**Table 2:** Effect of reaction time on the color of reduced-fat and reduced-sodium chicken meat batter gel with TGase.

Sample	L*	a*	b*
T1	81.67 ± 0.25 ^c^	1.86 ± 0.10 ^a^	12.39 ± 0.30 ^a^
T2	82.65 ± 0.32 ^b^	1.68 ± 0.04 ^b^	12.43 ± 0.40 ^a^
T3	83.25 ± 0.29 ^a^	1.49 ± 0.15 ^c^	12.57 ± 0.35 ^a^
T4	82.59 ± 0.45 ^b^	1.19 ± 0.11 ^d^	12.51 ± 0.33 ^a^
T5	82.18 ± 0.31 ^b^	1.05 ± 0.07 ^d^	12.64 ± 0.43 ^a^

T1: reaction at 4 °C for 0 h; T2: reaction at 4 °C for 6 h; T3: reaction at 4 °C for 12 h; T4: reaction at 4 °C for 18 h; T5: reaction at 4 °C for 24 h. Each value is the mean ± SE, n = 4. a–d Different parameter superscripts indicate significant differences (*p* < 0.05).

**Table 3:** Effect of reaction time on texture characteristics of reduced-fat and reduced-sodium chicken meat batter gel with TGase.

Sample	Hardness (N)	Springiness	Cohesion	Chewiness (N·mm)
T1	72.00 ± 1.39 ^e^	0.903 ± 0.008 ^b^	0.704 ± 0.011 ^a^	39.04 ± 0.96 ^c^
T2	74.47 ± 1.05 ^d^	0.910 ± 0.010 ^ab^	0.708 ± 0.016 ^a^	40.57 ± 0.49 ^c^
T3	81.09 ± 0.34 ^a^	0.922 ± 0.004 ^a^	0.711 ± 0.011 ^a^	43.41 ± 0.74 ^a^
T4	78.62 ± 0.97 ^b^	0.916 ± 0.009 ^a^	0.707 ± 0.022 ^a^	41.75 ± 0.60 ^b^
T5	76.80 ± 0.65 ^c^	0.912 ± 0.006 ^ab^	0.708 ± 0.009 ^a^	40.58 ± 0.58 ^c^

T1: reaction at 4 °C for 0 h; T2: reaction at 4 °C for 6 h; T3: reaction at 4 °C for 12 h; T4: reaction at 4 °C for 18 h; T5: reaction at 4 °C for 24 h. Each value is the mean ± SE, n = 4. a–e Different parameter superscripts indicate significant differences (*p* < 0.05).

**Table 4:** The initial relaxation time (ms) and peak ratio (%) of cooked reduced-fat and reduced-sodium chicken meat batter gel with different TGase reaction times.

Sample	Initial Relaxation Time (ms)			Peak Ratio (%)		
	T_2b_	T_21_	T_22_	P_2b_	P_21_	P_22_
T1	1.23 ± 0.14 ^a^	93.99 ± 6.33 ^a^	960.75 ± 33.93 ^a^	1.28 ± 0.11 ^a^	80.57 ± 1.11 ^e^	17.97 ± 1.21 ^a^
T2	0.97 ± 0.15 ^a^	80.31 ± 3.22 ^b^	850.96 ± 34.09 ^b^	1.22 ± 0.11 ^a^	83.90 ± 1.35 ^d^	14.81 ± 1.28 ^b^
T3	1.06 ± 0.27 ^a^	50.26 ± 1.71 ^e^	532.51 ± 18.16 ^e^	1.14 ± 0.10 ^a^	92.16 ± 0.47 ^a^	6.69 ± 0.51 ^e^
T4	1.05 ± 0.06 ^a^	65.21 ± 2.61 ^d^	634.19 ± 41.53 ^d^	1.17 ± 0.11 ^a^	90.02 ± 0.93 ^b^	8.81 ± 0.87 ^d^
T5	0.98 ± 0.12 ^a^	73.63 ± 2.60 ^c^	727.79 ± 25.70 ^c^	1.27 ± 0.16 ^a^	87.05 ± 1.39 ^c^	11.68 ± 1.38 ^c^

T1: reaction at 4 °C for 0 h; T2: reaction at 4 °C for 6 h; T3: reaction at 4 °C for 12 h; T4: reaction at 4 °C for 18 h; T5: reaction at 4 °C for 24 h. Each value is the mean ± SE, n = 4. a–e Different parameter superscripts indicate significant differences (*p* < 0.05).

**Table 5:** The formulations of reduced-fat and reduced-sodium chicken meat batters with different TGase reaction times.

Sample	Chicken Breast Meat (g)	Pork Back Fat (g)	Sodium Alginate Solution (g)	TG (g)	Reaction Time (h)
T1	200	20	20	1.86	0
T2	200	20	20	1.86	6
T3	200	20	20	1.86	12
T4	200	20	20	1.86	18
T5	200	20	20	1.86	24

Note: Added 40 g of ice water, 2 g of sodium chloride and 4 g of spices to all samples.

## Data Availability

The original contributions presented in this study are included in the article. Further inquiries can be directed to the corresponding author.
